# Running a clinical trial remotely: Lessons learnt from a decentralised multicentre randomised controlled trial evaluating a digital health intervention for Chronic Kidney Disease

**DOI:** 10.1371/journal.pdig.0001166

**Published:** 2026-02-20

**Authors:** Gurneet Kaur Sohansoha, Noemi Vadaszy, Ella C. Ford, Thomas J. Wilkinson, Matthew Graham-Brown, Alice C. Smith, Courtney J. Lightfoot

**Affiliations:** 1 Leicester Kidney Lifestyle Team, Department of Population Health Sciences, University of Leicester, Leicester, United Kingdom; 2 NIHR Leicester Biomedical Research Centre, Leicester, United Kingdom; 3 Department of Cardiovascular Sciences, University of Leicester, Leicester, United Kingdom; 4 School of Humanities and Social Sciences, Leeds Beckett University, Leeds, United Kingdom; 5 Diabetes Research Centre, University of Leicester, Leicester, United Kingdom; 6 Department of Renal Medicine, University Hospitals of Leicester NHS Trust, Leicester, United Kingdom; National Tsing-Hua University: National Tsing Hua University, TAIWAN

## Abstract

Decentralised clinical trials (DCTs) are a potentially efficient and cost-effective way of delivering research trials. My Kidneys & Me, a self-management digital health intervention for chronic kidney disease, was evaluated in a multi-centre randomised DCT (SMILE-K) (ISRCTN18314195). This study aims to evaluate recruitment outcomes and research staff experiences of delivering the SMIKE-K DCT, to inform the design of future DCTs. SMILE-K used fully remote trial processes, including online outcome measure collection. Recruitment and retention data were collected, including numbers invited, recruited, and completing outcome measures, and methods of invitation and consent. Quantitative data were analysed descriptively. Following trial recruitment, semi-structured interviews were conducted with research staff at external recruiting sites to explore their perspectives and experiences of remote trial processes. Qualitative data were analysed using thematic analysis. 420 participants were recruited to SMILE-K. The median time from expression of interest to consent was 1 day (range:0–100), and from consent to randomisation was 6 days (range:0–197). Thirteen research staff were interviewed. Six themes were identified: ‘discordance between perceptions and experiences of recruiting participants’, ‘reallocation of available resources across research studies’, ‘more environmentally friendly’, ‘onus on participants’, ‘engaging disadvantaged groups of participants’, and ‘future considerations to improve recruitment’. Results suggest that a DCT design can reach a high number of eligible participants. An invitation flyer via post after a remote clinical appointment was the most successful method of recruitment. Research staff felt DCTs provided opportunities for a diverse and representative population to participate and study procedures were environmentally friendly; however, consideration must be given to the factors that may affect recruitment and participation. Our research highlights a clear disparity between the expected recruitment rate and the reality of recruiting for DCTs, with research staff indicating they faced unanticipated challenges during the process. We outline factors for consideration when designing and delivering DCTs.

## Author summary

Decentralised clinical trials (DCTs), where some or all parts of the trial happen remotely, are believed to offer a practical approach to test interventions. Our team conducted a multi-centre randomised DCT that tested a digital health intervention. We used digital tools for trial processes and data collection. We interviewed research staff involved in the trial to learn more about their experiences of running a DCT. Our findings suggest that this type of approach has the potential to enable numerous people to take part. We found that the most successful route to invite was by speaking to them about it first, rather than directly sending letters out in the post. Using a DCT design was believed to provide an opportunity to recruit a diverse group of participants, reduce travel for participants, and be more eco-friendly. However, the research staff involved faced some challenges; the lack of direct communication was considered hard, and there were some misconceptions about who could and would be willing to participate, particularly regarding age and digital skills. Our findings have helped us understand more about how to best conduct DCTs and identified factors for consideration when designing future DCTs to improve trial delivery.

## Introduction

Chronic kidney disease (CKD) is a long-term condition associated with high morbidity and premature mortality [1] and has an estimated UK prevalence of ~5%–7% [2].In UK, 70% of National Health Service (NHS) expenditure is spent on patients with long-term conditions such as CKD [3]. With <1% of time spent in contact with healthcare professionals, many patients are expected to self-manage their condition [3]. Decentralised clinical trials (DCTs), where some or all trial activities occur remotely, are increasingly popular as they can reduce participant burden, increase trial accessibility and recruitment, and improve the generalisability of findings [4]. They may be a cost-efficient approach to delivering research trials [5,6]. Recent literature highlight that while DCTs have expanded rapidly, particularly since the COVID-19 pandemic and can offer promising opportunities to widen access and reduce research disparities, they also present new challenges related to data integrity, regulatory oversight, and equitable access [7]. Although DCTs offer potential benefits in reach and convenience, evidence on their effectiveness and data quality is limited [6]There may be particular value in using DCTs to pragmatically evaluate digital health interventions (DHIs).

Digital health interventions to deliver (health)care to and support self-management in people with long-term conditions have been the subject of considerable interest [8,9]. Despite evidence suggesting that technology-based interventions may improve clinical outcomes and disease management [10], DHIs supporting self-management and health-promoting behaviours in chronic kidney disease (CKD) are lacking. Systematic reviews and meta-analyses on DHIs have shown mixed or uncertain effects on outcomes such as physical activity, dietary adherence, medication management, and quality of life, with many studies limited by small sample sizes, short follow-up periods, and inconsistent outcome reporting [11–13]. Moreover, few interventions are grounded in behavioural theory or co-designed with patients, and most are developed for dialysis populations rather than individuals with early or moderate CKD [14,15]. Collectively, this highlights a clear gap in robust, theory-informed, and scalable DHIs that support long-term self-management and health-promoting behaviours across the CKD population.

To support self-management in people with CKD, we co-developed and tested ‘My Kidneys & Me’ an evidence- and theory-based self-management DHI that provides tailored interactive information and support to improve people’s knowledge, skills, and confidence in managing their CKD [16,17]. The clinical effectiveness of ‘My Kidneys & Me’ was demonstrated in a multi-centre randomised controlled trial (RCT) [17]. The trial testing the DHI had an adaptive design and was delivered as a DCT, with all trial processes conducted remotely [18].Given that the research staff responsible for clinical trial delivery are vital to the recruitment and retention of participants and correct implementation of trial protocols, their perspectives and experiences are invaluable to refine and improve the design and delivery of future DCTs.

### Aim

The aim of this study was to assess recruitment outcomes and explore research staff experiences in delivering the DCT, with the intention of informing the design of future decentralised clinical trials.

## Methods

The ‘Self-Management Intervention through Lifestyle Education for Kidney health’ (SMILE-K) trial was a mixed-method RCT with a pragmatic adaptive design and remote study processes [17]. This mixed-methods sub-study evaluated recruitment processes and metrics in the SMILE-K trial and explored research staffs’ experiences with delivering and recruiting to the SMILE-K. The sub-study was initiated during the conduct of the trial, when the research team identified emerging insights and recognised an opportunity to capture the perspectives of staff involved in trial delivery. Combining quantitative and qualitative approaches provided a richer and more practical understanding of how the SMILE-K trial was delivered and the recruitment processes used. Findings from the main SMILE-K trial are reported elsewhere [19]. Patient experiences of participating in a DCT are being evaluated separately.

### Ethics approval and consent to participate

This sub-study uses ethics from the SMILE-K trial (quantitative data from patient participants) and DIMENSION-KD study (qualitative data from research staff participants). The broader aim of DIMENSION-KD is to explore the perspectives and experiences of individuals who do not have CKD themselves but are affected by it through their professional roles, and in particular, their views on the design and delivery of lifestyle interventions for people living with CKD. The SMILE-K trial was fully approved by the Research Ethics Committee-Leicester South on the 19/11/2020 (reference: 17/EM/0357). All participants provided informed consent online. All participants were given the opportunity to ask questions before completing the consent process. The study was conducted in accordance with the Declaration of Helsinki. The trial is sponsored by the University of Leicester (rgosponsor@le.ac.uk). The study was prospectively registered as ISRCTN18314195 https://www.isrctn.com/ISRCTN18314195 on 18/12/2020. Participants in this sub-study were recruited as part of the DIMENSION-KD study. The study was approved by the Leicester Research Ethics Committee (24/05/2018, reference: 18/EM/0117) and prospectively registered as ISRCTN84422148 in June 2018 https://www.isrctn.com/ISRCTN84422148. The study was conducted in accordance with the Declaration of Helsinki and local and national ethical guidelines. All participants provided informed online consent.

### SMILE-K trial design

SMILE-K was a prospective, single-blinded, multi-centre RCT with a nested pilot, recruiting participants from 26 hospital sites providing kidney services across England, UK. All study procedures and processes were conducted remotely and digitally, partly due to the COVID-19 pandemic but also to pragmatically evaluate a DHI. A full description of the SMILE-K protocol, including full recruitment procedures and processes, has been published [20]. Key processes and procedures are summarised below.

### SMILE-K study processes and procedures

#### SMILE-K trial participants and recruitment.

Participants aged ≥18 years with CKD stages 3–4 (eGFR: 15–59 ml/min/1.73m^2^), not receiving kidney replacement therapy, were eligible to participate [17]. Eligible participants were identified by clinical teams and provided an invitation letter and flyer. Methods for providing study invitations/flyers were pragmatic and determined by individual research sites (e.g., hard copies given face to face or posted in the mail, or electronic copies sent via email). Interested potential participants emailed the study team, who then sent sequential emailed links: 1) a link to the online patient information sheet (PIS) and consent form, 2) a separate link to complete baseline outcome measures as a survey, including demographic data and health questionnaires (e.g., Patient Activation Measure (PAM-13) (primary outcome)).

**Fig 1:**
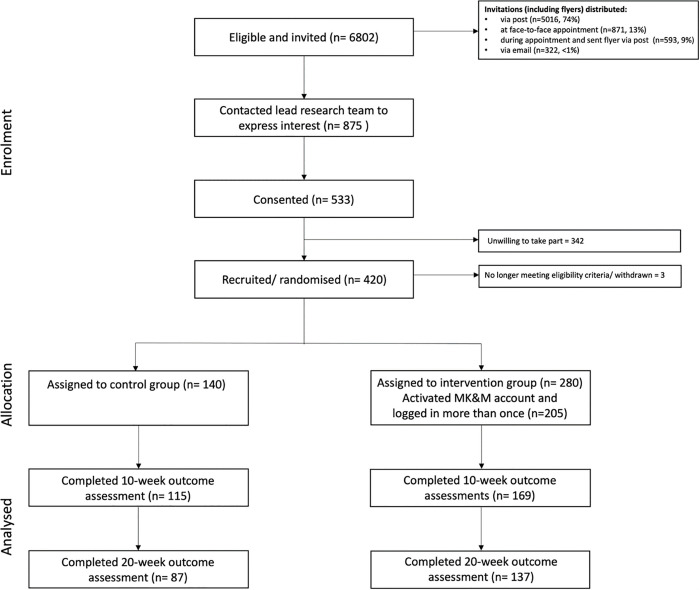
A flowchart depicting progress through SMILE-K trial phases Lightfoot, C.J., Wilkinson, T.J., Sohansoha, G.K. et al. The effects of a digital health intervention on patient activation in chronic kidney disease. npj Digit. Med.7, 318 (2024).

All surveys, forms and questionnaires were completed via JISC Online Surveys. Participants were randomised 2:1 to receive the MK&M DHI or continue usual care. Subsequently, links for outcome measure surveys were sent to all participants at 10-week and 20-week time points. Recruitment processes are summarised in Fig 1 (CONSORT flow diagram). If any surveys were not completed within 5 days of being sent, a reminder was sent.

### Recruitment metrics

Data were collected on the number of participants invited and recruited, and the number of participants who completed outcome measures at different time points. The methods of invitation letter distribution (e.g., in person, post, email), the number of potential participants that expressed interest, and the number of PIS sent, were recorded by the lead site from forms filled out by individual recruiting sites. The number of participants who consented to the study and the method of how they were approached were collected via an online survey. All data were handled by the lead site (Leicester, UK).

### Qualitative interviews

#### Participant selection and recruitment.

Research staff who were involved in delivering and recruiting to the SMILE-K trial, including principal investigators (PIs) and research staff from 25 external recruiting sites, were invited to take part in telephone semi-structured interviews to explore their views and experiences of the study processes. Participants were recruited as part of the DIMENSION-KD (ISRCTN84422148) study, a prospective observational cross-sectional study. To be eligible, participants had to have been involved in setting up and/or delivering the SMILE-K trial. Potential participants were invited to be interviewed by the lead research team, and sent a PIS, via email. If there was no response within two weeks, a follow up email was sent. No further contact was made if no response after the follow-up email. If interested in taking part, participants completed an online consent form and a demographic questionnaire. A suitable time for an interview was then arranged.

Participants were purposively selected based on their site’s recruitment performance and engagement with the lead research team to ensure diversity of experiences. The participants’ job titles, years of experience, and involvement with the SMILE-K trial were considered during the selection process for the interview. Participants who had difficulty with recruitment and struggled to reach their target recruitment numbers, along with those from sites that experienced minimal recruitment issues, were chosen to provide insights to help the lead research team understand the challenges encountered and identify strategies that worked, as well as areas for improvement in future trials.

### Sample size

A target sample size of ten was chosen to reflect diversity in views and experiences within the available time and resources, in line with accepted sample sizes for similar qualitative studies [21]. The sample size was subject to change depending on the richness of participant data [22].

### Interview procedure

Interviews were conducted via telephone at a single time point, following the cessation of study recruitment. One researcher (GKS) conducted the interviews, supported by an experienced qualitative researcher (CJL). Interviews were audio-recorded and transcribed verbatim by a professional transcriber. Researchers kept a personal reflective logbook during data collection and analysis.

### Topic guide

Interview topic guides explored participants’ views on the acceptability of the trial, intervention, and outcomes. The topic guide was developed and adapted using the SMILE-K patient topic guide used by the Leicester Kidney Lifestyle Team and was piloted on initial participants before being finalised. The topic guide included questions about participants’ experiences and the SMILE-K trial, including their thoughts on SMILE-K, how they found running a DCT, what the barriers and facilitators were, and what could have been improved. The topic guide also explored participants’ thoughts on the study process, including their thoughts about online surveys, how participants were approached, and positive or negative aspects of the study process. Specific questions about study documents including distribution methods of the PIS and the flyer, were explored and how these affected staff and participant experience.

## Data analysis

Data on SMILE-K participants are expressed as mean and standard deviation (SD), or median and range (n-n), unless otherwise stated. Descriptive and frequency analysis was used using SPSS 28 (IBM SPSS Statistics).

Interview data were managed and coded by hand. Data were analysed using thematic analysis [22,23]. GKS familiarised herself with the complete data set through listening to audio recordings and annotating transcripts, and independently identified initial codes using an inductive approach. Samples of transcripts were independently coded by CJL. Following confirmation of similar code derivation, the remaining transcripts were coded by GKS. Potential themes were created through identifying relationships between codes and collating them to form over-arching concepts. GKS and CJL reviewed and refined themes, and definitions of themes were agreed upon. The COREQ guidelines were followed when reporting the methods.

## Results

### SMILE-K trial participant characteristics

Twenty-six hospital sites in England recruited participants to SMILE-K between May 2021 and December 2022. The 420 recruited participants had a median age of 60 years (range:20–88), 60% were male, and 92% were White British. The mean eGFR was 38.8 (±18.8) ml/min/1.73 m^2^). SMILE-K participant characteristics can be found in the supplementary material S1 Table.

### Methods of invitation to SMILE-K

A flowchart of participation can be found in Fig 1. 6802 invitations were issued to potential participants: 74% (5016) by mail, 13% (871) in-person, 9% (593) by mail following remote consultation, and 4% (322) by email. Of those invited, 13% (875/ 6802) expressed interest, of which 60% (533/875) consented.

Of those who consented, 49% (265/533) were initially invited via mail, 23% (120/533) in-person, 19% (103/533) via mail following remote appointment, and 1% (5/533) via email. Of those who consented, 80% (420/533) completed the baseline survey and were subsequently randomised. 67% (282/420) completed the 10-week survey, and 53% (224/420) completed the 20-week survey.

The median time from expression of interest to consent was 1 day (range: 0–100), and consent to randomisation was 6 days (range: 0–197).

### Summary of qualitative interviews

Based on their site’s recruitment performance, 30 research staff across the 25 recruiting sites were invited to participate, with 16 responding. Thirteen consented to take part and were interviewed. Interview participant characteristics are displayed in Table 1. Interviews lasted an average of 30 minutes (range: 20–47 minutes). Six overarching themes were identified. Exemplar quotes are shown in Table 2.

**Table 1:** Interview participant characteristics’.

Participant Number	Gender	Ages (years)	Ethnicity	Job Title	Years in position
DCT01	Female	56	White British	Research Project Manager	Not stated
DCT02	Female	49	Indian	Clinical Research Nurse	10
DCT03	Female	49	White British	Clinical Research Assistant	Not stated
DCT04	Female	Not stated	White British	Clinical Research Nurse	Not stated
DCT05	Female	57	White British	Clinical Research Nurse	34
DCT06	Female	30	White and Black Caribbean	Research Practitioner	Not stated
DCT07	Female	42	White British	Research Assistant Practitioner	Not stated
DCT08	Male	59	White British	Research Assistant	7
DCT09	Female	65	White British	Research Nurse	45
DCT10	Female	51	White British	Senior Research Nurse	24
DCT11	Female	44	White British	Principal Investigator	19
DCT12	Female	45	Indian	Clinical Trials Assistant	16
DCT13	Female	55	White British	Principle Investigator	30

**Table 2:** Themes and exemplar quotes.

Themes	Exemplar quotes
Discordance between perceptions and experiences of recruiting participants	*“I thought we would recruit a lot more people! No, no, no other expectations really. I just was surprised by how difficult I did find it to get people to actually consent to the study” – Clinical Research Assistant* *“So, it’s nice at the end of the month when you guys send the information to say that’s great, I have rung 58 people and it shows that I’ve got 40 consented, so it is pretty good” – Research Practitioner* *“It was harder than I expected. I thought, we all thought we would just get our, you know, I think in the first month I contacted something like 35 patients and got nobody. So, I was really really shocked”- Research Nurse*
Enhancing the efficiency in reallocation of available resources across research studies	*“Like I say I have found it pretty straightforward. I have to say it’s been one of the easier studies that I’ve been involved with, cause its sort of almost run itself. I think compared to other trials it’s felt, it’s felt easier, it’s felt less labour intensive.” – Principal Investigator* ***“*** *You know, like certain things can be done from different offices, so certainly in terms of your resources, like talking about us in our department, there isn’t a need for people to be so close to each other or, you know, meeting in person. It can all be done on Teams. So, I think our department has embraced that kind of way of working as well” – Research Project Manager* *“I’ve quite enjoyed it personally. I’ve had quite a lot of control. Certainly, with my electronic site file, I think it’s worked out well for me, because I’ve got all the information here just in front of me.” Research Project Manager*
More environmentally friendly	*“You ask them to sign up to it, they fill in the consent form and it’s digital, hit send and it uploads and saves all these bits of paper and millions of trees that we use all over the place” - Research Nurse* *“This group of patients, already have a lot of hospital visits. And I think with petrol going up and everything… Like that, they are quite reluctant to have to come in for extra appointments, because they’re already coming quite a lot” – Research Assistant* *“Oh, it’s much better than storing; we need to cut down on the amount of paper that we go through” - Research Nurse*
Onus on participant	*“So, I suppose, you know, you might argue maybe there’re people who’ve been less likely to sign up because the onus has been on them very much” - Senior Research Nurse* ***“*** *So, I think, I think the online stuff is, is good and we need to move more towards that kind of You know, if there’re opportunities to do things remotely I think that that’s really useful, it’s easier for patients, it’s, it’s not as onerous for them to take… To either be recruited or take part in. So, I think that that’s really useful, particularly from an exercise point of view, you know, for them not to have to turn up and do an exercise class or whatever, you know, that’s, you know, I think that was probably something that appealed to the patients and what have you.” - Research Nurse* *“I suppose, you know, the patients who, they have to be able to engage with the platform, so if they can’t sign an online consent form then they may not be appropriate for the type of intervention.”- Principal Investigator* *I found that if we send anything out in most people may think about it, may say they’ll do it, put it in the top drawer, forget about it, or as soon as they get in put it in the bin.”- Research Assistant*
Engaging disadvantaged groups of participants	*“I had this preconceived idea that it would be the younger people that would be interested in it. And that didn’t turn out to be the case; there was a lot of older people that were really interested. Just shows you mustn’t prejudge anybody” - Research Assistant* *“I screened a 91-year-old gentleman, and I did think to myself oh, he’s not going to be interested in this. He’s not going to use computers, but I thought well no, cause he’s eligible. And you can’t not offer the trial. And I rang him and he was completely computer literate and he was really up for it and really interested, yeah” - Research Assistant* *“So just people’s ease and ability and… Want to engage with digital resources, online resources. I guess there’s some inequality type things, you know, access to a computer. Access to a smartphone that’s able to sort of, you know, engage properly with it, people who are older who may, you know, and we generalise, obviously lots of older people are now fantastically tech savvy, but there will be people who aren’t, plus things like eyesight and ability to sort of physically use resources due to, you know, joints and things like that I guess.”- Principal Investigator* *“And then the one sort of one that I hadn’t really thought about, one of my GP colleagues had mentioned, was around people who can’t, can’t read and can’t write. But especially those who, other languages, so I suppose at the moment it’s a resource in English, so translating into different languages will obviously help the people that can read and write in those languages, but I guess then you’ve got that other group of people who can’t read and write English and can’t read or write in another language, and I guess it’s something else needed for them.” - Principal Investigator*
Future considerations to improve recruitment	*“I think we would have liked, looking back - and I think [Nurse] would agree - the ringing up of the patients would have made a difference and that’s how we would run it differently. In reality, which was our intention at the start, to have that sort of personal contact” - Research Nurse* ***“*** *I think maybe we could be, for future, we’ve got more people in the team now. Maybe a follow-up phone call after they got the information. Because it might refresh people’s memory, you know, if they had got the information but they weren’t sure about it.” – Research Assistant* *“it’s important, to have little bit more personal involvement, if we could, if it was viable, that we could help the trial go a little bit better.” - Research Assistant* *“But I think sometimes they need follow-up, otherwise some patients are forgetful and they just… But when we are calling they’re oh yeah, I just forgot to read it, I will do it today.” – Research Assistant*

### Discordance between perceptions and experiences of recruiting participants

Research staff expressed how they expected recruiting to a DCT would be easy and attractive to potential participants. Some research staff discussed how DCTs can reach a greater proportion of individuals, but research staff also experienced challenges and struggles facing recruitment. Research staff reported large discrepancies between the number of participants screened and the number who consented. It was expressed that some of the recruitment challenges that the research staff faced could be prevented by having follow-up calls with participants to further discuss the trial.

### Enhancing the efficiency in reallocation of available resources across research studies

The DCT processes were described as “straightforward” and “easy to do”, freeing up time and effort for more resource-intensive research studies (e.g., COVID-19 studies). Research staff also discussed the benefits of an e-site file and how easy it is to access study information, therefore, saving time when storing and looking for study documents compared to traditional trials.

### More environmentally friendly

Research staff felt that DCTs were more environmentally friendly compared to traditional clinical trials. The reduced need for participant travel, electronic documentation for participants (PIS, information sheets, consent forms) and research staff (e-site file) reportedly helped to reduce the trial’s carbon footprint and paper use. Having the PIS online was perceived to reduce the length of the document by only providing essential information within the text and then having links available for the participants to read further information if they wished.

### Onus on participant

Research staff described how the remote online processes were driven by the participant, as participation in the trial was dependent on individuals contacting the research team directly and communicating via email. It was believed that this may have put potential participants off taking part, resulted in a lack of engagement, or disadvantaged certain groups of patients (i.e., digital poverty).

### Engaging disadvantaged groups of participants

The wide inclusion criteria of the trial were perceived to be inclusive, with the potential to engage a wide range of participants. Whilst the study design was thought to potentially disadvantage or exclude some participants (e.g., those with poor digital literacy, lack of digital access, language barriers and disabilities), it was argued that if participants were unable to sign an online consent form, then a trial evaluating a DHI may not be appropriate for them. Research staff described how their preconceptions regarding potential participants’ age and digital literacy influenced whether they would approach the potential participant or not to discuss the trial. They acknowledged the need to not assume that individuals may not be interested in the trial and approach all eligible participants as they may wish to participate and benefit from the study. Research staff discussed how they were surprised at the desire, willingness, and ability of older individuals to engage in the trial.

### Future considerations to improve recruitment

Research staff highlighted specific strategies that could be used to support the delivery of and could increase recruitment to future DCTs. Many felt that adopting a more personalised approach to recruitment, by phoning participants as a reminder, offering further explanation, and highlighting key aspects of the study, could engage more individuals and potentially support disadvantaged groups. In addition to recruitment, several research staff reflected on the importance of follow-ups to maintain participant engagement throughout the study as this could remind participant to complete study tasks and enhance retention.

## Discussion

**Fig 2:**
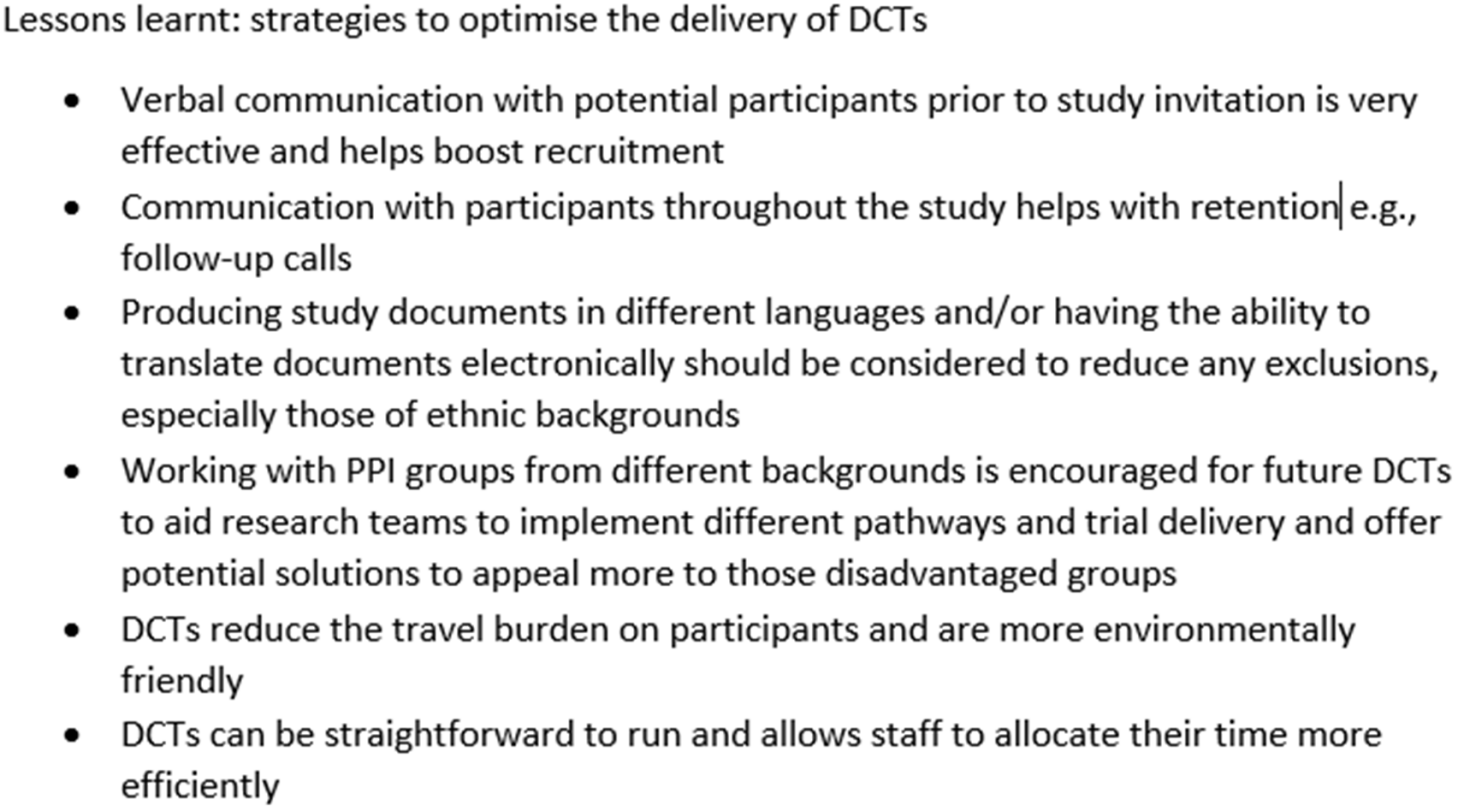
Lessons learnt: strategies to optimise the delivery of DCTS.

The findings from this mixed-method study demonstrate that recruitment to a DCT provides the opportunity to reach a high number of eligible participants and reduces some barriers associated with traditional trials. However, this DCT was perceived to create different barriers to participation and may exclude people who experience digital poverty (limited access to digital devices, internet connectivity, or digital literacy) or linguistic barriers [24]. A summary of the lessons learnt can be found in Fig 2.

Of those invited to take part, only 13% expressed interest in participating in the trial, and 7% were recruited, representing a difference between those interested in taking part and those who actually participated in the trial. It is not clear from this study why this discrepancy exists and there is a lack of research to support this; however, existing studies indicate that participants may not fully understand the consent form or the details of the study, which could be a deterrent [25,26]. Some potential reasons suggested by research staff were that the onus is on the participants to contact the research team, and potential participants may forget about the study after the initial invitation. Following up with participants and having a more personalised approach to recruitment were suggested as potential methods for better trial engagement. Research staff also reflected on participant follow-up as an important factor for retention and to be considered in future trials. They noted that some participants could have benefit with reminders to complete study tasks as poor retention may be partly due to participants forgetfulness or competing priorities, research staff felt that consistent contact and reminders could have improved participant retention. Other research has highlighted that maintaining contact is vital for participant retention [27].

Most trial invitations were issued by post with no prior approach or explanation, which resulted in a markedly lower response rate than invitations issued after discussion with a healthcare professional, either remotely or in person. This suggests that verbal communication about the study is more effective, and supports the research staff’s perspectives that adopting a more personalised approach, with increased communication throughout the study, would better facilitate recruitment and retention in the trial. This is consistent with other studies, which have demonstrated that direct human contact is generally more effective in enhancing participants’ understanding of study documents and the overall study objectives [28]. This is also in keeping with other research that suggests maintaining contact with participants throughout the study with follow-up calls is essential for retention [27] and that having verbal communication with a trusted healthcare professional before the study has a positive effect on recruitment [29]. There is a lack of evidence to support which recruitment routes have the best recruitment rate, but it is important to maintain different options to engage different groups of patients. Whilst not a strategy we used, recruitment via social media platforms has been suggested as an additional successful recruitment strategy [8].

This DCT recruited a wide range of participants with an age range of 20–88 years, of which 58% were male, suggesting the strategies used supported the recruitment of a diverse population in relation to age and gender. The proportion of males recruited aligns with findings from other studies [30]; however, a broader age range was recruited in this study. There was a disproportionate representation of White ethnicity, with 92% of the recruited participants in the study being White British; this is higher than the population in England where 82% of the population in England are White (2021 census data) [31], and those generally recruited to trials - the NIHR reported that 86% of participants recruited into RCTs in 2022 were White [32]. The low levels of non-White participants recruited into this study, whereby only 8% of the recruited population representing diverse ethnic backgrounds may be a result of limited efforts to engage specific groups during recruitment, thus more targeted recruitment strategies are crucial for future trials.

The qualitative findings highlight that participant documents and the DHI evaluated in the trial were all in English. In addition, one of the trial exclusion criteria was an “insufficient command of English or any other precluding factors that prevent the ability to give informed consent or comply with protocol”. Collectively, these disadvantaged individuals who cannot read and/or write English from participating. Individuals who experience language and communication barriers often find trial participation difficult [33]. Individuals from ethnic minority backgrounds also frequently experience underrepresentation in RCTs as a result of language barriers and cultural differences [34]. Thus, adapting and improving the design of DCTs is required to meet the needs of these groups who are likely to benefit greatly by taking part in DCTs to better their health [35]. Adaptations could include: producing study documents in different languages, having the ability to translate documents electronically, and/or offering audio versions. Working with PPI (Patient and Public Involvement) groups from diverse backgrounds could aid research teams in implementing different pathways and trial delivery and offer potential solutions to appeal more to those disadvantaged groups.

This DCT was perceived by research staff to be more environmentally friendly than traditional clinical trials by reducing the trial’s carbon footprint as a result of reducing participants’ travel burden, paper use and consumption. It is estimated that traditional NHS clinical trials account for 6% of the UK’s total carbon footprint [36]. A DCT approach was felt to be a way of reducing this footprint and was felt to be of considerable benefit compared to traditional trials. Other research has also found that participants’ expressed likelihood to enrol in a DCT to lessen travel burden and that DCTs reduce travel barriers [8,37].However, due to the remote nature of the trial, eliciting and maintaining interest from potential participants was considered challenging. A lack of verbal communication with participants, misconceptions regarding age and digital literacy, and barriers to using the trial intervention, such as low digital literacy, access to technology and language barriers were discussed by research staff in our study. Potential study participants who may have wanted to engage and would have benefitted from the trial and its intervention may not have been able to participate due to limited access to digital resources or poor digital literacy. To understand and effectively use DHIs, a certain level of digital literacy is required; thus, poor digital literacy can be an important barrier to participation [38]. Solutions for participants who experience digital poverty should be considered to ensure DCTs using online processes reduce the number of potential participants excluded, such as setting up local hubs in areas to allow access to digital devices and having digital champions to help support the use of digital programmes. Enhancing digital literacy through education and support can empower individuals to engage effectively with DCTs(34). Research staff interviewed in this study reported having preconceived ideas that DHIs would only interest younger patients; however, the trial recruited a range of ages, including a greater proportion of the older population. Decentralised trials and DHIs appear to have the ability to be inclusive and provide the opportunity to engage with disadvantaged groups of people, however, further improvements and adaptions are crucial to reduce the number of potential participants that are excluded due to language barriers and access to digital platforms.

The SMILE-K DCT was perceived to be more challenging to recruit than traditional trials, evidenced by the high number of participants invited, but the proportionally low uptake. However, after increasing communication efforts with potential participants, recruitment was believed to be easier as a result of answering queries. The participants drove the remote nature of this trial and its initial processes, which could result in poorer engagement. A more personalised approach and better communication were considered to support a higher recruitment rate for future DCTs, but could potentially reduce the generalisability of study findings as the trial design is then less pragmatic, (i.e., real-world engagement would be expected to revert to being lower once the additional trial support and processes for recruitment were removed if they were not available outside the trial). Most interviewed research staff highlighted that the trial was more environmentally friendly than traditional trials, as all files are stored electronically, and study-related documents can be stored/ hosted online and sent via email. Hospital or traditional clinic visits are also not needed in DCTs, resulting in less travel for participants’ when compared to traditional trials, which could also reduce logistical barriers that may prevent someone from enrolling or staying with a trial and also supporting trials to be more environmentally friendly.

### Recommendation to improve the delivery of DCTs

From our findings, it is important to consider enhanced and effective communication with potential participants to improve study recruitment and retention. This could include phoning participants at various time points across the study to help maintain interest and provide additional information if needed. Study documents, such as the PIS, could be condensed and written in clearer lay terms to not discourage participants from being involved in DCTs, or have supporting audio/video information. Effective strategies to engage more with disadvantaged groups, such as minority ethnic groups and people with low digital literacy or lack of access to digital resources, must be developed and tested to ensure we are not widening health inequalities. Working with PPI groups from different backgrounds is essential to improve the delivery of DCTs or to help engage more with disadvantaged groups.

### Strengths and limitations

The main limitations of the SMILE-K are documented elsewhere [19], but in summary, the trial population was predominantly White British, therefore, findings may not be generalisable to those from minority ethnic groups. Also, potential participants who were not able to read English, or experienced digital poverty, were unlikely to participate due to the DCT and DHI design. It could be argued that traditional trial processes would have better engaged participants with lower health literacy and from a more diverse range of backgrounds; however, this was not possible due to the necessary remote design of the trial. Reducing some barriers associated with traditional trials, such as not having hospital visits, reduced the travel burden for participants. In addition, a reduction in paper usage and electronic documentation resulted in the trial being more environmentally friendly. The qualitative interview findings helped to further explain these limitations by highlighting practical challenges encountered during trial recruitment and delivery. Research staff reflected on barriers, including digital poverty and linguistic challenges, that may have disproportionately excluded certain groups, reinforcing concerns about limited diversity. Interviews also revealed site-level differences in resources and communication that influenced recruitment success, factors not captured by quantitative data alone. This contextual insight emphasises the need for tailored strategies to overcome these barriers in future trials. The main strengths of the sub-study were that there was a wide range of participants that took part in the interviews from different ages, years of experience and job titles, as they all had different experiences of the trial from these variants. However, there was only one male who took part in the interviews. Another limitation is that the participants were interviewed after the study had finished, which meant the recall of the study process might not have been as strong as it would have been if interviews had been carried out during the study.

Another limitation observed with DCTs, particularly in lifestyle-based research, is the challenge of evaluating the quality of lifestyle outcomes. In the SMILE-K study, while we placed considerable emphasis on physical activity, we encountered difficulties in accurately capturing this behaviour. Similarly, although improving physical function was one of our objectives, we were constrained to relying on subjective assessments. This issue extends to other lifestyle factors, such as sleep, where objective measurement would ideally be preferred but is not feasible due to the nature of a DCT. A potential solution to navigate this limitation could involve the use of accelerometers for more precise tracking of physical activity or using video assessments to evaluate physical function, which could offer a more objective approach, this approach is shown in similar research [39].

## Conclusion

Experiences from the SMILE-K trial suggest that DCTs may provide a pragmatic and widely accessible approach to the evaluation of interventions, particularly DHIs. However, efforts to engage more with disadvantaged groups are essential. Our findings show a notable disconnect between anticipated outcomes and actual experiences encountered when recruiting for DCTs, with many research staff reporting facing challenges throughout the process. Our findings highlight areas which require consideration to improve the design and delivery of DCTs to maximise access to their potential.

## Supporting information

S1 Checklist COREQ guidelines. (PDF)

S1 Table SMILE-K participant characteristics. (DOCX)

S2 Table CONSORT 2010 checklist. (DOCX)

S1 Data Quantitative data. (XLSX)
