# Supplementary material for: Running a clinical trial remotely: Lessons learnt from a decentralised multicentre randomised controlled trial evaluating a digital health intervention for Chronic Kidney Disease
Source: PLOS Digit Health. 2026 Feb 20;5(2):e0001166. doi: 10.1371/journal.pdig.0001166 (PMC12923010; doi:10.1371/journal.pdig.0001166)
Supplement: S1 Table — (DOCX) [file pdig.0001166.s002.docx]

**S1 Table**. SMILE-K participant characteristics

|  | **All**  **(n=420)** | **Intervention (n=280)** | **Control**  **(n=140)** |
| --- | --- | --- | --- |
| Age, years | 59.8 (±13.4) | 59.4 (±13.6) | 60.7 (±12.8) |
| Sex male, n (%) | 250 (60%) | 161 (58%) | 89 (64%) |
| **Ethnicity**, n (%) |  |  |  |
| White British | 384 (92%) | 254 (91%) | 130 (93%) |
| Other White | 14 (3%) | 8 (3%) | 6 (4%) |
| South Asian | 10 (2%) | 8 (3%) | 2 (1%) |
| Black | 5 (1%) | 4 (1%) | 1 (1%) |
| Other ethnic group | 7 (2%) | 5 (2%) | 1 (1%) |
| **Education**, n (%) |  |  |  |
| None | 8 (2%) | 4 (1%) | 4 (3%) |
| Primary school | 2 (1%) | 1 (<1%) | 1 (1%) |
| High school | 82 (20%) | 1 (<1%) | 1 (1%) |
| College | 133 (32%) | 90 (33%) | 43 (31%) |
| University | 138 (33%) | 87 (32%) | 51 (37%) |
| Other trade/ vocation qualification | 51 (12%) | 39 (14%) | 12 (9%) |
| **Occupation**, n (%)  Retired  Employed  Unemployed  Other (disabled, long-term sick leave) | 183 (45%)  208 (51%)  12 (3%)  4 (1%) | 115 (43%)  145 (54%)  6 (2%)  4 (1%) | 68 (50%)  63 (46%)  6 (4%)  0 |
